# Storage conditions differentially alter the human tooth enamel proteome

**DOI:** 10.3389/fdmed.2025.1666534

**Published:** 2025-11-14

**Authors:** Hakan Karaaslan, Baptiste Depalle, Felicitas B. Bidlack

**Affiliations:** 1 ADA Forsyth Institute, Somerville, MA, United States; 2 The Forsyth Institute, Cambridge, MA, United States

**Keywords:** enamel, proteome, storage, tooth, proteomics

## Abstract

**Introduction:**

Exfoliated or extracted human teeth are increasingly used as accessible records of health history, biomarkers for predicting health risks, or analyzed for organic components to uncover the mechanisms of atypical development or disease. During formation, dental enamel incorporates and preserves biological information through mineralization, thereby creating a fossil-like record within the tooth structure as it reaches a mineral content of over 95% by weight. The key to unlocking this record depends not only on using appropriate analytical methodologies, but also on how storage conditions affect the original biogenic information.

**Methods:**

To investigate the effect of storage conditions on enamel proteome, human third molars were collected upon extraction and stored for 4 months under four commonly used protocols in dental research: (1) at −80°C; (2) in 70% ethanol (EtOH) at room temperature (RT); (3) air-dried (Air) at RT; and (4) in phosphate-buffered saline with sodium azide at RT.

**Results:**

Mass spectrometry-based proteomic analysis identified 454, 460, 232, and 221 proteins in the −80°C, EtOH, Air, and PBS groups, respectively. Enamel-specific proteins, such as amelogenin, ameloblastin, enamelin, kallikrein-related peptidase-4, and matrix metalloproteinase-20, were identified across all conditions, although their relative abundances varied depending on storage conditions. In addition, the preservation of specific protein families varied depending on the storage conditions.

**Discussion:**

Although −80°C storage remains the gold standard for preserving organic material, storage in 70% EtOH at RT produced comparable proteomic results. This suggests that ethanol-based storage protocols may serve as a more practical alternative, easier to implement for sample collection, and help provide consistency in enamel research. Our findings underscore the importance of both storage methods and standardized protocols in enamel proteomics, as they help avoid bias in protein detection and facilitate comparisons of datasets between studies.

## Introduction

The composition of tooth enamel and dentin, including the abundance of proteins, is critical for dental health and tooth properties (1–3). The high mineral content in tooth enamel confers remarkable durability to teeth. It also preserves the incremental growth structures and entraps the ephemeral organic matrix, which mediates biomineralization (4–6). With advancements in analytical methods, teeth and tooth enamel are appreciated in scientific research as archives of biological information about development, environment, identity, behavior, as well as revealing prenatal and postnatal exposures, determining sex, and capturing snapshots of the proteome at the time of tooth development (7–14). In addition, the analysis of the organic portion in tooth enamel is key to understanding the causes and mechanisms of dental defects and to developing strategies to improve tooth enamel properties and resistance to decay (15, 16). However, the reliability of information derived from the proteomic analyses of the organic material within enamel greatly depends on its preservation under *ex vivo* conditions until the time of analysis.

The effects of storage conditions on the chemical composition and physical properties of dental enamel have previously been demonstrated to alter its mechanical properties (17–20). It has also been shown that modifying the protein matrix of enamel significantly affects the outcomes of biomaterial studies aimed at enhancing restorative outcomes (21, 22). However, no systematic comparisons have been published on how storage conditions affect the proteome of enamel.

In this study, we investigate how common storage conditions for extracted teeth affect the outcomes of enamel proteomic analyses. Addressing this question is essential for establishing standardized and reliable protocols for storing extracted or exfoliated teeth in future studies. Furthermore, understanding of how specific storage conditions affect the preservation of different protein families is critical for identifying potential biases in proteomic datasets and guiding protocol development for studies targeting specific proteins.

## Materials and methods

### Collection of teeth and storage conditions

This study was reviewed and approved for exemption by the Institutional Review Board of ADA Forsyth Institute (19-03e). We analyzed the human tooth enamel proteome after subjecting it to four common storage conditions for a period of 4 months. We used unerupted, healthy human maxillary third molars with fully developed roots. Teeth were immediately transferred to dry ice upon extraction, transported to the laboratory, and processed on the same day. Each tooth was quartered, and one quarter was assigned to each of the four storage conditions. This approach enabled us to subject the same tooth to different storage conditions and pool several teeth for a given storage condition, thereby averaging biological variability in protein matrix composition across individuals.

**Figure 1:**
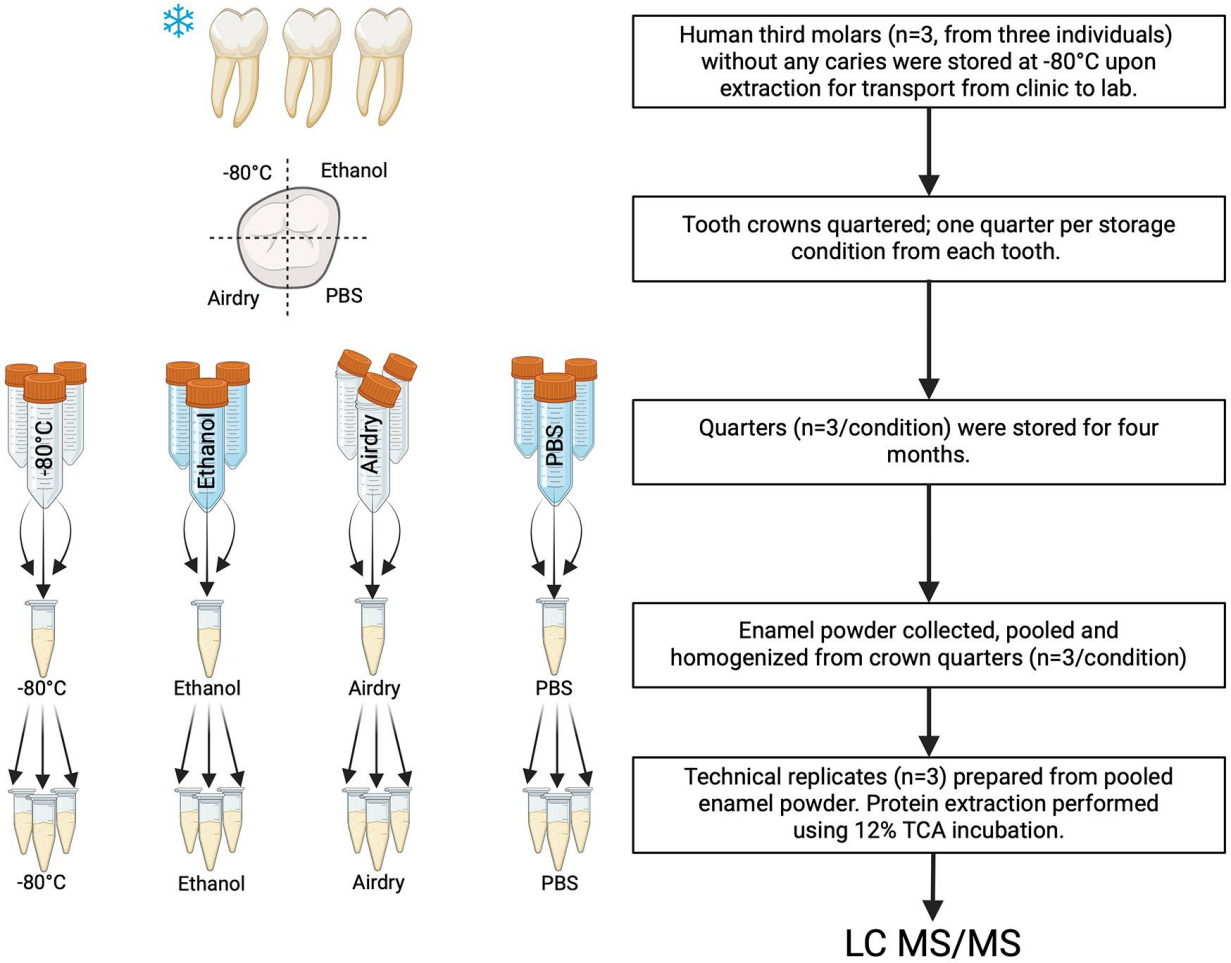
The experimental design of this study involved using pooled and homogenized enamel powder obtained from three teeth per condition, from which technical replicates were prepared for mass spectrometry analysis. Each tooth used for the study was divided into quarters, and each quarter assigned to one of the four different conditions. This procedure averaged biological variability and allowed us to determine the effects of the experimental conditions. Proteins were extracted using 12% trichloroacetic acid (TCA) incubation and analyzed using mass spectrometry.

The following common storage conditions were tested: −80°C; 70% ethanol (EtOH) solution at room temperature (RT); air-drying (Air) at RT; and phosphate-buffered saline (PBS) with sodium azide at RT (*n* = 3 per condition). Each tooth quarter was placed in a 50 mL tube and subjected to one of the storage conditions for 4 months (Figure 1). Air-dried samples were briefly dried using lab wipes and were stored in an open tube. No protease inhibitor was added to the storage solutions because of the limited penetration through enamel.

### Enamel collection and protein extraction

At the end of 4 months, all samples were cleaned by brushing with deionized water (diH_2_O) for 1 min using a disposable toothbrush. Approximately 50 µm of the superficial enamel layer was removed using slow-speed burs to remove surface contamination. Enamel powder was collected from the outer two-thirds of the full enamel thickness. Powder collected from the crown quarters was pooled for each storage condition and was homogenized for 5 min using a vortex mixer and sample rotator, switching every 30 s. Three technical replicates were prepared from the pooled powder for each condition.

Enamel samples were processed using a slightly modified version of our previously reported protocol to improve our final yield (23, 24): 20 mg of enamel for each replicate was incubated in 2 mL 12% trichloroacetic acid (TCA) with a protease inhibitor cocktail (cOmplete™ Mini; Roche Diagnostics, Germany) for 4 h at 4°C. Protease inhibitors are only included in the protein extraction step once the experimental period is over, in order to better simulate the storage conditions. Individual tubes were agitated using a sonicator bath for 1 min every 30 min during the TCA incubation (25). They were then centrifuged at 20,000*g* for 5 min at 4°C. Protein pellets were washed twice with acetone (−20°C, 13,000*g*). Final pellets were dried at RT for 30 min, then resuspended in an 8M urea solution, followed by adding 50 mM ammonium bicarbonate buffer, and stored at −20°C until gel electrophoresis.

### Mass spectrometry analysis

Extracted proteins were loaded into 10% Mini-Protean TGX Precast protein gels (Bio-Rad Laboratories, Hercules, CA, USA) and were run for 10 min (Supplementary Figure S1). Gel pieces, including the whole half-run lane, were cut and subjected to in-gel trypsin digestion. Peptides were subjected to electrospray ionization and then entered into an LTQ Orbitrap Velos Pro ion-trap mass spectrometer (23). Peptides were detected, isolated, and fragmented to produce a tandem mass spectrum for each peptide. Peptide sequences were identified by matching protein databases with the acquired fragmentation pattern by the software program Sequest. All databases contained the reverse form of all sequences and the data were restricted to a peptide false discovery rate in the range of 1%–2%. Proteins were included in the analyses only when identified by at least two non-overlapping peptides with more than nine amino acids, following HUPO standards (26).

Data were processed and normalized using DEP package on R (27). Data imputation for the missing datapoints was performed using the MinProb function and the Shannon diversity index was calculated using the vegan package on R. Protein classification was performed using PantherDB. Data visualization was performed using ggplot on R, Prism 9 (Graphpad Software Inc., San Diego, CA, USA) and InteractiVenn (28). Biorender.com was used for schematic representation.

## Results

**Figure 2:**
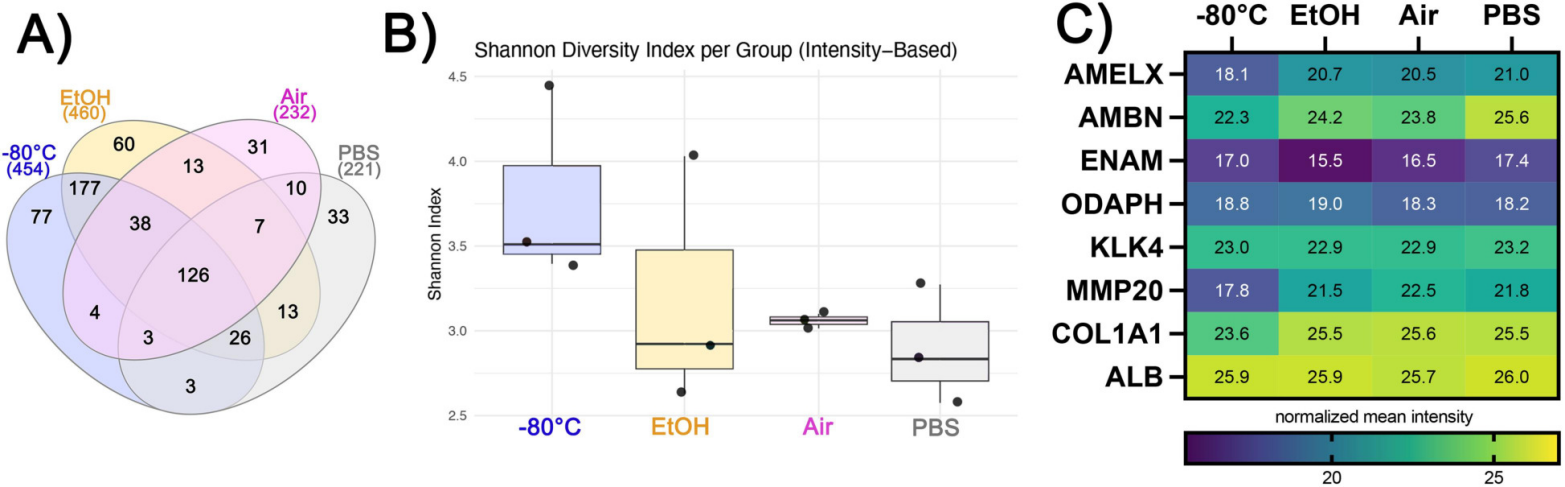
**(A)** Venn diagram showing numbers of unique and shared proteins across four different storage conditions. **(B)** Shannon diversity index values based on intensity values of the proteins. **(C)** Heatmap showing mean relative abundance of enamel matrix proteins ameloblastin (AMBN), enamelin (ENAM), and amelogenin (AmelX), commonly studied proteins, such as odontogenesis-associated phosphoprotein (ODAPH), kallikrein-related peptidase-4 (KLK4), matrix metalloproteinase-20 (MMP20), Alpha-1 chain of type-1 collagen (COL1A1), and serum albumin (ALB).

The number of identified proteins is highest in EtOH, similar in −80°C conditions, and less than half in Air and PBS. We identified 454, 460, 232, and 221 proteins in the conditions of −80°C, EtOH, Air, and PBS, respectively (Figure 2A). The number of unique proteins identified only in a particular condition was 77 for storage at −80°C and 60 for EtOH samples, with 177 common proteins in these experimental groups that were not identified in the PBS or Air groups. In contrast, PBS and Air samples had 33 and 31 unique proteins, respectively. Based on the Shannon diversity index (Figure 2B), storing enamel at −80°C resulted in the highest diversity of the extracted proteome, while the diversity of the proteome was lower in EtOH and PBS stored samples, albeit similar in those two groups. In contrast, the highest homogeny between replicates was seen in Air stored samples.

The relative abundance of enamel-specific proteins differs between storage conditions. We identified enamel-specific proteins in all samples, including amelogenin (AMEL), ameloblastin (AMBN), enamelin (ENAM), and odontogenesis-associated phosphoprotein (ODAPH) (Figure 2C) with the following relative abundances: AMEL was highest in PBS and lowest in −80°C; AMBN was highest in PBS and lowest in −80°C; ENAM was highest in PBS and lowest in EtOH; and ODAPH was highest in EtOH and lowest in PBS. The enamel proteases kallikrein-related peptidase-4 (KLK4) and matrix metalloproteinase-20 (MMP20) were also identified and quantified. The highest relative abundance of KLK4 was in PBS and the lowest was in EtOH and equal in EtOH and Air, while the highest relative abundance of MMP20 was in Air and was lowest when stored at −80°C (Figure 2C).

We also identified non-enamel-specific proteins that are usually detected in enamel proteomic studies and considered biologically relevant. Representing collagen, Collagen 1 alpha chain was identified in all samples; it had the highest relative abundance when stored air-dried and the lowest abundance when stored at −80°C (Figure 2C). Serum albumin (ALB) had the highest abundance when the samples were stored in PBS, while the lowest abundance was observed when they were stored in Air (Figure 2C). All proteins and their normalized relative abundances are listed in Supplementary Sheet 1. Proteins that are significantly different across different storage conditions can be seen in Supplementary Figure S2 and Sheet 1.

**Figure 3:**
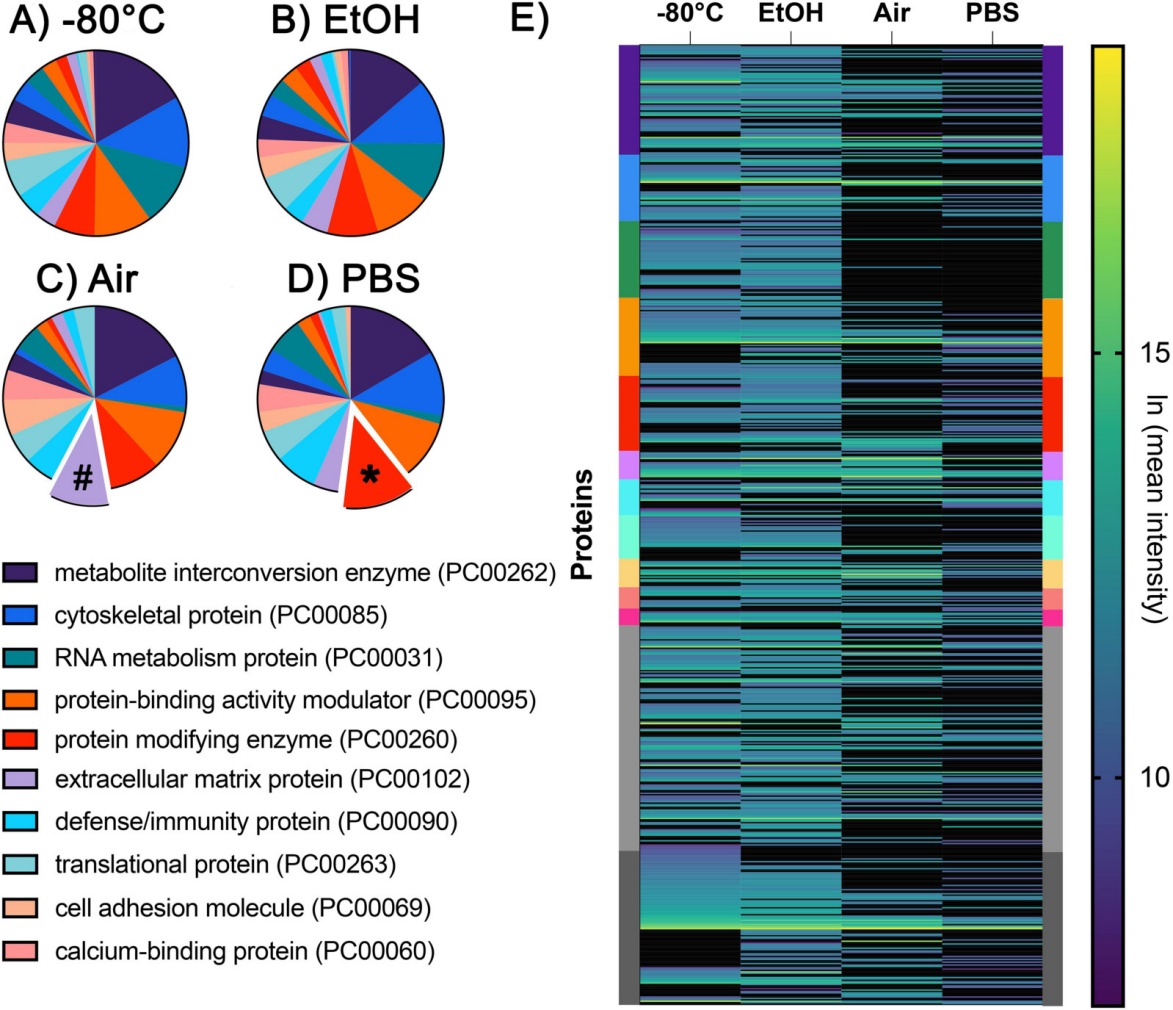
Pie charts illustrating the top 10 protein classes identified in the **(A)** −80°C, **(B)** EtOH, **(C)** Air, and **(D)** PBS storage conditions. Protein classes were determined using the PantherDB and ranked according to the number of proteins identified in each class. The symbols (#) and (*) indicate protein classes ranked higher in the Air **(C)** and PBS **(D)** groups. RNA metabolism proteins were among the top 10 protein classes in the **(A)** −80°C and **(B)** EtOH groups, but not in the **(C)** Air or **(D)** PBS groups. **(E)** Heatmap showing the relative abundance of proteins across each experimental group. Individual proteins are represented as single lines and color-coded according to the same PantherDB protein classes shown in the protein classes in panels **A–D**.

The order of protein classes, if ranked by the highest number of proteins, differs between samples and their storage conditions. A comparison of protein classes with the highest number of proteins between samples of different storage conditions is shown in Figures 3A–D (29). The top protein class was metabolite interconversion enzyme (PC00262) in all storage conditions. The closest match between the distribution of the top 10 protein classes was between −80°C and EtOH conditions (Figures 3A,B); however, the following differences remained: defense/immunity class (PC00090) was missing in the top 10 in EtOH, while this is the top ninth category in the −80°C group; the extracellular matrix protein group (PC00102) was the seventh category in EtOH-stored samples but was the 10th protein category in −80°C samples. Interestingly, the category of extracellular matrix proteins was the second-highest category in the Air samples (number sign in Figure 3C) and ranked ninth in PBS storage conditions (Figure 3D).

Some protein classes were more conserved in EtOH and PBS. The protein-modifying enzyme group (PC00260) was the second-highest category in PBS samples (asterisk in Figure 3D), while it was the fifth highest category in the other three conditions. The RNA metabolism protein (PC00031) category was the third highest protein category among the −80°C and EtOH groups, but it was not among the top 10 in the Air and PBS groups. Finally, the individual proteins and their relative abundances are shown in Figure 3E. Each protein is represented as a line, and the protein classes that they belong to are indicated with their color on the left and right *Y* axis (Figure 3E).

## Discussion

Various methods are used to store extracted teeth before analysis, depending on the availability of resources in a clinical setting and the practicality of sample storage and transport. For dental educational purposes, where no downstream chemical analyses are planned, the CDC recommends autoclaving or immersion in 10% formalin for 2 weeks (30). However, these methods significantly alter the mineral and organic components of the tooth. If proteins are to be studied, autoclaving will not only destroy infectious agents but also alter the organic matrix of the dental tissue, and formalin fixation will result in the cross-linking of molecules, affecting protein extraction from tooth samples. Therefore, less aggressive methods are preferred for studying the organic matrix of dental tissues in proteomics. Saline, ethanol, sodium hypochlorite, or thymol solutions are commonly used for storage, whereas freezing is typically preferred when maximum preservation of the organic phase is required (31–33).

Freezing at −80°C is typically considered the gold standard for storing biospecimens, as both enzymatic and non-enzymatic degradation of proteins are significantly reduced at this temperature (34). However, studies have shown that biomolecules can still undergo alterations at −80° C, potentially affecting the organic components, including proteins and nucleic acids, when stored for extended periods (35–37). In our study, the protein composition was comparable between the −80°C and EtOH groups, with similar overall protein composition and relative abundance of most relevant proteins, based on PantherDB protein classes. Therefore, storage in 70% ethanol at room temperature offers a practical and effective alternative to −80°C, especially in clinical settings where immediate freezing is often not feasible. Ethanol storage could also reduce the shipment costs in multicenter studies and provide favorable antibacterial properties.

Our results demonstrate better conservation of the proteome in the EtOH and −80°C groups compared to the PBS and Air groups. Although the −80°C provided the most diverse proteome when compared to all other conditions, there are notable differences in the preservation of specific proteins. For instance, the relative quantities of enamel-specific proteins such as MMP20 and ameloblastin were lowest in the −80°C group compared to all three conditions (Figure 2B). The relative abundances of collagen and KLK4 in air-dried samples were comparable to those stored at −80°C, and amelogenin levels were even higher. Similarly, air-dried samples showed an increase in the overall percentage of the extracellular matrix protein category (PC00102), while the other categories were similar to those in the −80°C group (Figure 3). These findings suggest that extracellular matrix proteins are more resilient to protein degradation due to environmental changes; however, it is unclear whether and to what extent residual enzymes contribute to this degradation.

Saline solutions, such as Hank's Balanced Salt Solution and PBS, are commonly used in both clinical and laboratory settings to preserve cell viability on tooth surfaces, including periodontal ligament cells. However, in this study, the PBS and Air groups had the lowest total number of proteins, which was less than half the number detected in the −80°C and EtOH groups. The temperature of the solutions in our study (RT) could have affected the results, and storing PBS at 4°C rather than RT might increase the yield. Interestingly, relative abundances of key proteins were lowest in PBS, even when compared to Air.

We observed a high abundance of proteins that are not specific to enamel (e.g., from blood and surrounding tissues) in all groups, as might be expected in unerupted, extracted teeth. These non-enamel-specific proteins have been previously reported (38–40). The high level of these proteins in the EtOH and Air groups in this study suggests either their deeper penetration into enamel or the preservation of non-enamel-specific proteins under these conditions.

In the case of proteins considered exogenous, such as serum albumin, the storage medium becomes especially important when these proteins are studied and their role is considered in the pathophysiology of developmental dental defects (15).

A limitation of our study is the short-term storage of all samples at −80°C for transport. Although all samples were subjected to the same step, hereby setting a baseline, the freezing followed by another storage condition might result in a difference in preservation compared to the absence of freezing. Another limitation is that we did not test the variability of protein content between the four quarters of the unerupted teeth, assuming that protein composition would be sufficiently similar in all four quarters of a single tooth crown, based on our understanding of the processes and patterns of tooth crown formation (41, 42). Further investigation looking into the effect of storage conditions could also focus on the post-translational modifications of the enamel proteome.

Our findings can inform future studies on selecting the optimal sample storage medium, taking into account both logistical considerations and specific research questions. Studies focusing on the albumin content in hypomineralized enamel may require a different storage method than those interested in MMP20 levels in developing enamel. Different storage conditions not only significantly alter the abundance of specific protein groups but also make it difficult to compare data between studies. For example, the difference in protein content in enamel between studies can be attributed to the varying storage conditions for the analyzed teeth (25, 40).

## Data Availability

The mass spectrometry proteomics data have been deposited to the ProteomeXchange Consortium via the PRIDE partner repository with the dataset identifier PXD070154.

## Supplementary material

Supplementary Figure S1 Proteins were extracted from 20 mg of enamel for each replicate and were loaded to 10% TGX gels. Gels were run for 10 min to reduce background noise. Two-inch-long gel pieces were then cut and subjected to trypsin digestion.

Supplementary Figure S2 Differential enrichment analysis of the proteins was performed using the DEP package on R. Volcano plots showing the proteins that are significantly different between conditions: **(A)** 80°C vs. PBS, **(B)** EtOH vs. PBS, **(C)** EtOH vs. Air, and **(D)** Air vs. PBS. Significance: adjusted *p*-value <0.05, log2FoldChange >1. There were no significant differences in comparisons −80°C vs. Air and −80°C vs. EtOH. The adjusted *p*-values and the fold changes for all comparisons are listed in Supplementary Sheet 1.

Supplementary Sheet 1 List of all the proteins and their intensity values across all conditions and the results of the differential enrichment analysis can be found in separate sheets inside the document. Common proteins that are significantly different in more than one comparison are also listed separately.
