# Supplementary figures and images for: Storage conditions differentially alter the human tooth enamel proteome
Source: Front Dent Med. 2025 Nov 14;6:1666534. doi: 10.3389/fdmed.2025.1666534 (PMC12660288; doi:10.3389/fdmed.2025.1666534)

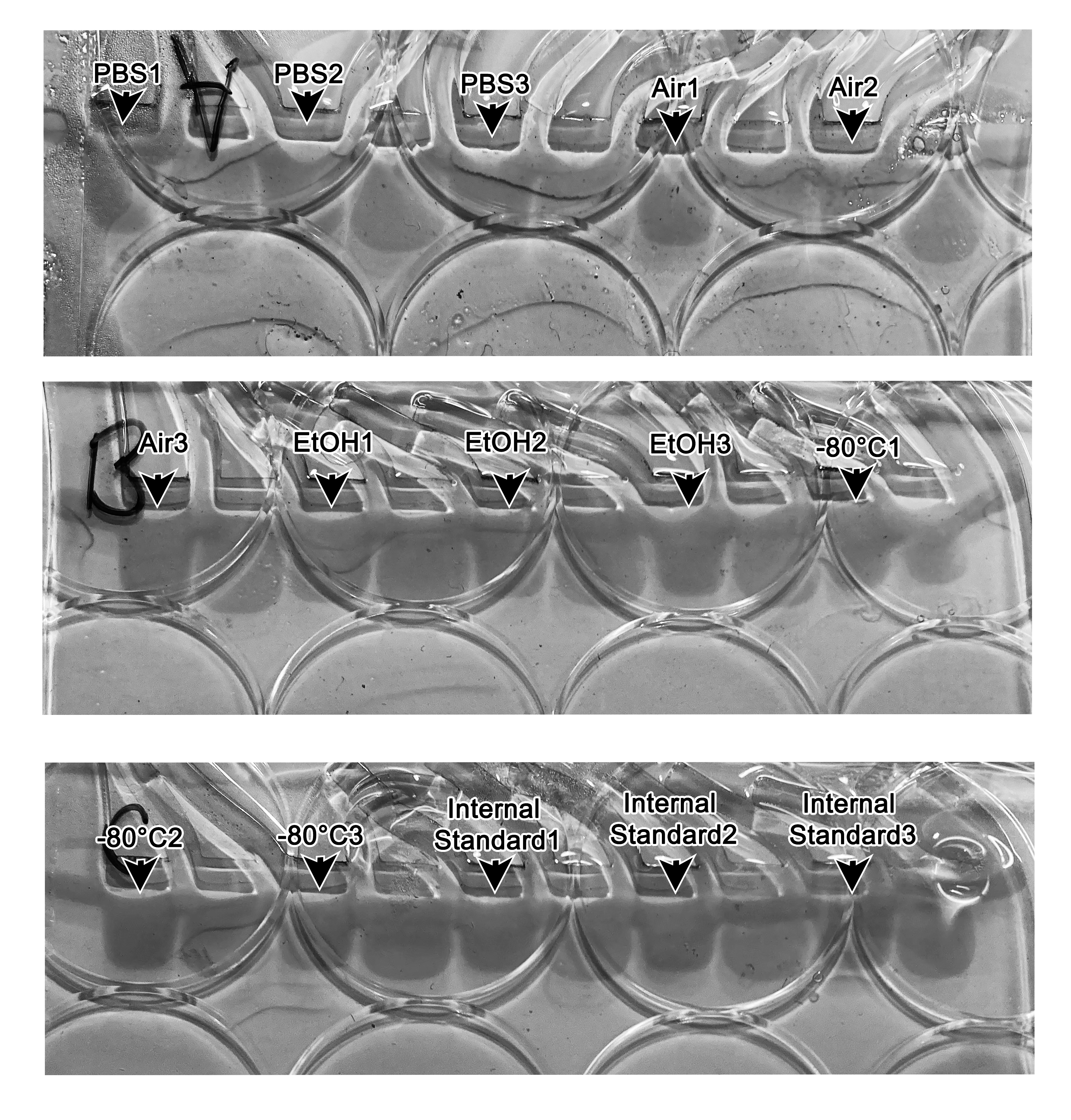

Supplement: Supplementary Figure S1 — Proteins were extracted from 20 mg of enamel for each replicate and were loaded to 10% TGX gels. Gels were run for 10 min to reduce background noise. Two-inch-long gel pieces were then cut and subjected to trypsin digestion. [file Image1.png]

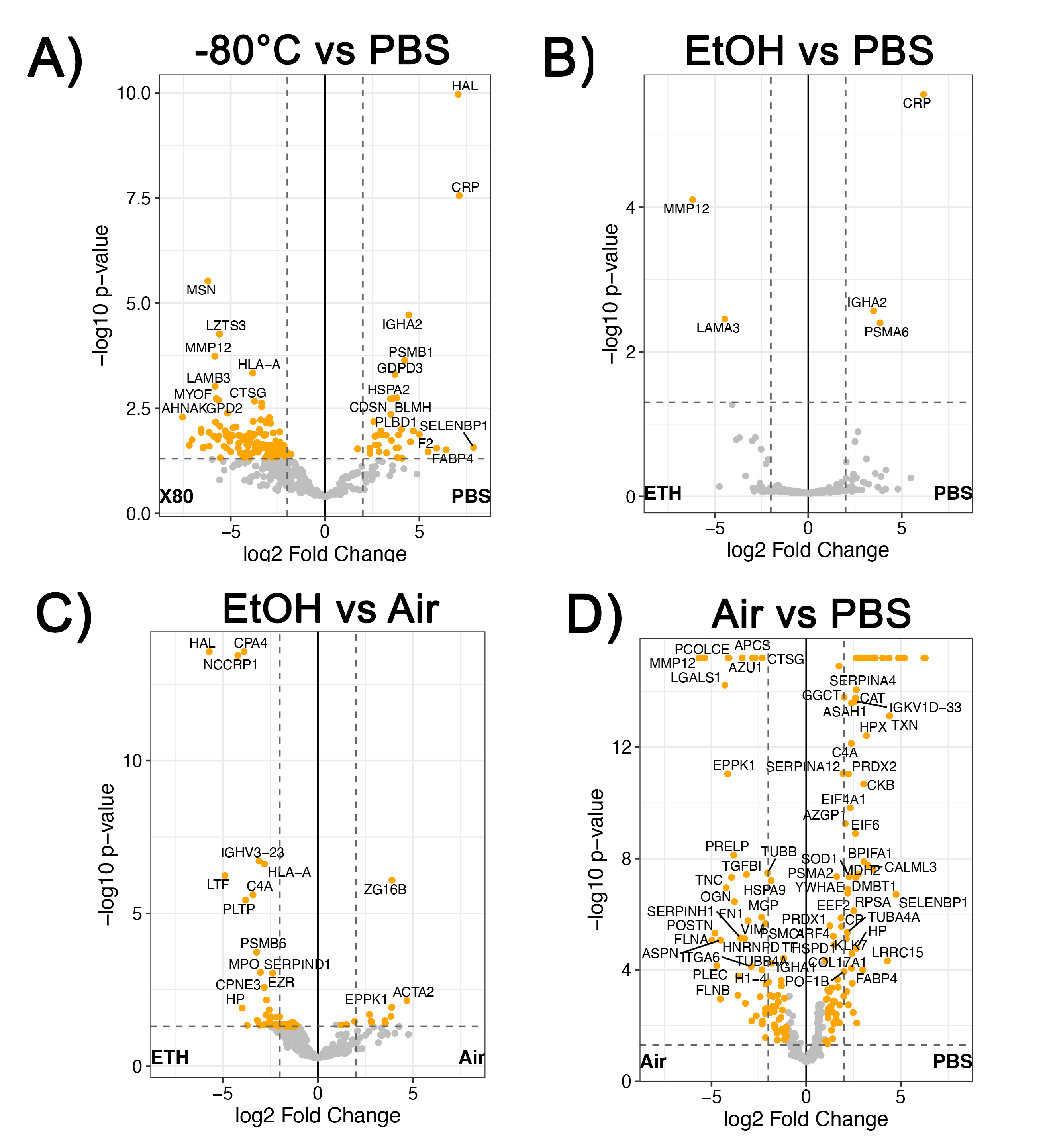

Supplement: Supplementary Figure S2 — Differential enrichment analysis of the proteins was performed using the DEP package on R. Volcano plots showing the proteins that are significantly different between conditions: (A) 80°C vs. PBS, (B) EtOH vs. PBS, (C) EtOH vs. Air, and (D) Air vs. PBS. Significance: adjusted p-value <0.05, log2FoldChange >1. There were no significant differences in comparisons −80°C vs. Air and −80°C vs. EtOH. The adjusted p-values and the fold changes for all comparisons are listed in Supplementary Sheet 1. [file Image2.png]
